# Infant eye and head movements toward the side opposite the cue in the anti-saccade paradigm

**DOI:** 10.1186/1744-9081-3-5

**Published:** 2007-01-17

**Authors:** Atsuko Nakagawa, Masune Sukigara

**Affiliations:** 1 Graduate School of Humanities and Social Sciences, Nagoya City University, 1, Yamanohata, Mizuho-cho, Mizuho-ku, Nagoya 467-8501, Japan

## Abstract

**Background:**

The anti-saccade task, when people must respond in the direction opposite to a visual stimulus, has been used as a marker of operation of the frontal cortical oculomotor area. However, early development of oculomotor control has been little studied with the infant anti-saccade paradigm, and a few studies did not recognize anti-saccades in infants in light of the results of adult anti-saccade. Since the characteristics of infant eye movements are little known, applying the criteria used in adult study is by no means the best way to study infant anti-saccade. As it is indicated that coordinated eye and head movements often enable infants to control the direction of their gaze, head movements should be examined as an infant orienting response. The aim of this study was to address how infants used eye and head movements during the anti-saccade paradigm. To distinguish infants' responses, we also investigated eye and head movements during a task for an inhibition of return. Inhibition of return, in which delayed responses occur in the direction to which attention had previously been oriented, has been thought to mark activity of the superior colliculus. Since the superior colliculus is thought to develop much earlier in life than the frontal lobes, we thought it useful to compare these task performances during infancy.

**Methods:**

Infants were divided into three groups according to age. Anti-saccade and inhibition-of-return tasks were given. Their eye and head movements during tasks were independently recorded by the corneal reflection method in the head-free condition.

**Results:**

Younger infants tended to initiate eye movement less than older ones in both tasks. In the anti-saccade task, responses opposite to the cue tended to show longer latency than responses to the cue. Infants made faster responses toward the side opposite the cue when it was to the right than when it was left of fixation. Regarding the comparison of responses toward the side opposite the cue between two tasks, the leftward eye movement was faster than the leftward head movements in the inhibition-of-return task, while no difference of latency was observed between eye and head movements in the anti-saccade task. A qualitative analysis of the trajectory of these responses revealed that head movement trajectories were steeper in the anti-saccade than in the inhibition-of-return task.

**Conclusion:**

Younger infants move head and eyes together, with head movements frequently starting first. On the other hand, both the leftward latency difference between eye and head and gentle trajectories of head in inhibition of return indicate that eye movements are more predominant over head movements in the inhibition-of-return task than in the anti-saccade task. This would suggest an earlier developing inhibition-of-return mechanism.

## Background

A crucial marker of eye movement is the ability to suppress saccades toward a suddenly appearing peripheral stimulus (pro-saccade), while making a saccade in the opposite direction instead (anti-saccade). In an anti-saccade task, subjects are instructed not to look at a flashed cue, but to make a saccade in the opposite direction [1]. That requires the willful inhibition of a strong drive to reflexively orient one's gaze to an abrupt visual stimulus. Using pro- and anti-saccade tasks, Munoz et al [2] revealed that children and adults diagnosed with attention deficit hyperactive disorders (ADHD) have great difficulties in suppressing unwanted saccades and voluntarily controlling their fixation behavior. They pointed to how the known fronto-striatal pathophysiology producing the symptoms of ADHD can damage the development of oculomotor control. There is a growing interest in oculomotor control in developmental disorders.

**Figure 1:**
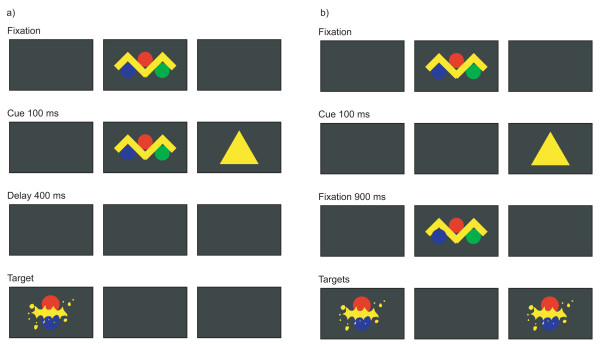
Stimulus sequence used in a) task of anti-saccade and b) task of inhibition of return.

Both Johnson [3] and Scerif et al. [4] tested infants' ability to inhibit saccades with an infant anti-saccade paradigm. Infant saccades were investigated by manipulating the spatial relationship between central and peripheral stimuli and the location where attractive stimuli appear. A similar presentation sequence (Figure 1a) was used in the present study. Giving verbal instruction to a young infant to look to the side opposite the cued side is impossible. Instead, one must encourage them to look at the second stimulus (the target) more than the first one (the cue) by making the second stimulus more attractive than the first one. After a number of such trials, Johnson [3] observed the decrease in responding to the cue, which predicted the appearance of attractive stimuli at a contralateral location. However, he concluded that 4-month-olds did not produce anti-saccades toward the target location in light of the results of adult anti-saccade (e.g., [5]). Scerif et al. [4] also did not recognize anti-saccades in infants, as they classified subjects' responses following the criteria used in adult study [5]. Thus, previous infant studies used adult criteria to classify infant responses in the anti-saccade task.

However, saccade characteristics themselves in young infants have been little studied systematically [6]. For example, as a methodological issue, it is not easy to restrict head movement and calibrate each subject, both of which are requisite for quantitative eye movement study. Thus, since the characteristics of infant saccades were little known, applying the criteria used in adult study was not by any means the best way to examine infant anti-saccade. Or infant responses during the task should be investigated before applying the criteria or being classified. While neither Johnson [3] nor Scerif et al. [4] recognized "anti-saccade" in early infants, by 4 months of age, infants can reportedly be trained to make saccades at particular spatial locations [7,8].

In a few cross-sectional studies [9-11], pro- and anti-saccades of subjects more than 5 years old have been examined in the general head-restrained condition to cancel out the effects of head movements. The characteristics of the saccades in the head-free condition may vary according to head movements. For example, a primate's saccades occurring in a head-free condition showed slower velocity or a depressed velocity-amplitude slope, relative to saccades occurring with the head fixed [12]. In order to differentiate between head-fixed and head-free saccadic eye movements, they have been termed eye saccades and gaze saccades, respectively.

Meanwhile, little is known about the infant head-free saccadic eye movements. Infant head movements have been mainly examined during visual pursuit in tracking of visual stimuli in order to study development of eye-head coordination [see [13]]. Johnson [3] and Scerif et al. [4] investigated infant oculomotor control with no specific concern as to its relationship with head movement. Although Regal et al. [14] noted that coordinated eye and head movements often enable infants to control the direction of their gaze, no one has studied how head movements are used by infants during an anti-saccade paradigm.

Until now we have attempted to quantify infant eye movements in the head-free condition, measuring eye and head movements independently at the same time [15]. In the present study, in applying our quantitative method, we examine the infant look toward the second stimuli (target) in the absence of response toward the first stimuli (cue) in an anti-saccade task (Figure 1a). These responses to the target were compared with their responses to the cued side. In each trial we measured both eye and head latencies, respectively. In their infant study regarding the coordination of eye and head movement in order to capture peripheral visual stimuli, Regal et al. [14] pointed out that the pattern according to which infant head movement precedes their eye movement is not found in adults.

Moreover, to distinguish infants' responses toward the above-mentioned non-cued side in the anti-saccade task, we also compared them with their responses during the task for an inhibition of return. The function of inhibition of return is to bias the subject to orienting toward novel objects and locations and away from previously inspected ones [16]. To be specific, in a spatial cuing paradigm for inhibition of return (Figure 1b), peripheral cues facilitate the processing of targets at cued locations for approximately 300 ms. However, with longer latencies between cue and targets, a spatial cue draws attention to that location reflexively, inducing an inhibition of saccades toward the cued location. In that covert method, a peripheral cue is presented so briefly that a saccade is not initiated. Therefore, inhibition of return seen from a covert paradigm using spatial cuing can also be called a reaction toward the opposite side from where the cue stimulus is given. However, these responses in the inhibition-of-return task are thought to be exogenously-driven automatic responses and to mark activity of the phylogenetically ancient superior colliculus [17]. On the other hand, those in the anti-saccade task are thought to be endogenously controlled and to require more computational resources [3]. To our knowledge, no one has yet made a quantitative comparison of eye and head movements during anti-saccade versus inhibition-of-return tasks. We want to see if the inhibition of return shows signs of maturity well before the anti-saccade task, since it is based on an older neural system which develops earlier.

The main purpose of the present study is to determine how infants used eye and head movements toward the side opposite the cued location, and to examine the characteristics of such movements during an anti-saccade task. Since anti-saccade task has emerged as an important tool for investigating not only normal brain function, but also dysfunction in various disease conditions [18], the early development of responses during that task is worth studying. Preliminarily, we also attempt to conduct a quantitative analysis of amplitude, velocity and latency of infant eye and head movements, in the head-free condition, during anti-saccade and inhibition-of-return tasks.

## Methods

### Subjects

Twenty-nine infants, ranging in age from 3 to 11 months (mean = 7.6 months, median = 7 months), were recruited through local maternity groups. All gave informed consent from their parents before the experiments. The study was approved by the Ethics Committee of Nagoya City University (No. 2) and accorded with the ethical standards specified in the 1964 Declaration of Helsinki. Infants were divided into three groups by age: eight 3- to 5-month-olds (4 male, 4 female), ten 6- to 8-month-olds (4 male, 6 female), and eleven 9- to 11-month-olds (6 male, 5 female). Criteria for admission into the study were: no known birth defects or other kinds of complications, full term (more than 37 weeks gestation), and normal birth weight (2500 g–4000 g). The data from another 4-month-old, two 5-month-olds, two 9-month-olds, and one 11-month-old were excluded because they were able to complete less than half of the inhibition-of-return or anti-saccade task trials. And one 11-month-old was dropped because he refused application of a sticking plaster to his forehead, which is explained in the following eye movement section. Even if infants completed enough trials, at times, we entirely failed to track the eye or head movements by the X-Y tracker as in the following section. Thus, owing to the insufficiency of the contrast in video images of infant faces, the X-Y tracker could not detect the brightest (or darkest) portions of the CCD sensor. Therefore, the data from one 5-month-old and two 6-month-olds were also excluded.

Each session, which consisted of inhibition-of-return and anti-saccade tasks, was scheduled for approximately 30 min during the infants' most alert time of day. If the infant was in a bad mood or not alert, the session was rescheduled. Upon arrival at the session room, the experimenter explained the general procedure while a research assistant handed the infant some warm-up toys to play with. After the infant seemed adjusted to both the room and the research assistant, the infant and mother were escorted to a semi-dark area surrounded by a blackout curtain. After completing one of two tasks, the infant and mother were escorted outside of the semi-dark area and the infant was soothed by the mother or the research assistant. Then they were taken back into the semi-dark area for the other task. During the experiments, the mother, though out of sight, was never far from the infant. At the end of the session, the mother was given the Infant Behavior Questionnaire-Revised (IBQ-R Japanese version) and asked to complete and return it.

### Eye movement recording

**Figure 2:**
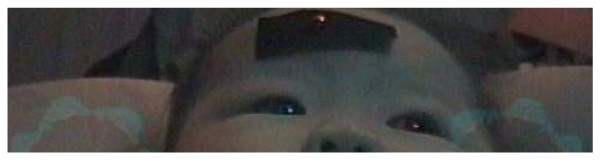
Recorded video image of 6-month-old infant, in which the visual stimuli were superimposed. To measure head movements, a small chrome steel ball bearing was affixed to the forehead using a black-colored sticking plaster.

The infant sat in an experimental baby chair 65 cm away from the color monitor of an AV tachistoscope (IS-702) in a semi-dark area. The experimenter outside the semi-dark area monitored the subject's eye movements through a low-angle CCD near-infrared video camera (ELMO CN43H) set in front of the infant, and controlled the stimulus presentation on the monitor by means of a microcomputer (FMV-S167). The stimuli presented were superimposed synchronously on video images of the eye movements by a digital image processor (FOR-A, MF-310) and recorded on videotape (SONY DSR-11), which was then used for off-line video coding (Figure 2). The central fixation stimuli appeared on the color monitor, while the peripheral stimuli were reflected in a first-surface mirror on the left or right side to maintain an appropriate distance from the central fixation of approximately 30 deg. Since head movements seldom occur in response to stimuli of target eccentricities of less than 20 deg [12], localization at the above eccentricity (30 deg) could be accomplished with saccadic eye and head movements.

Eye movements were recorded by the corneal reflection method. To measure infant head movements, a small chrome steel ball bearing (4.75 mm) was stuck to around the middle of the subject's forehead (Figure 2) and its reflected image was also recorded (SONY DSR-11). Beams of invisible infrared light (LED: SLR-938C) were directed at the subject's eye from the upper right. The reflected images of corneas and the chrome ball bearing were caught by a near infrared CCD camera (Hamamatsu Photonics, C3077-78) which was set up on the subject's lower left (see Additional file 1). The TV signal was digitized to a two-dimensional scale by an XY-tracker unit (Hamamatsu Photonics, C3162) off-line. This digitizer converted some of the brightest (or darkest) portions of the CCD sensor to digitized data with an absolute accuracy of 5118 × 3864 pixels at each 33.3 ms of sampling time. The performance of the left eye was analyzed.

### Calibration

An exact method of calibration has not yet been found. Since it is difficult to communicate verbally with infants, they can not be expected to follow orders involved in a calibration procedure. However, we applied the experimental tentative calibration method of Koga et al. [15], which employs the analysis of distribution of data of the landing position for gaze and head to determine the most likely frequent landing position. This approach relied on the eye and head movements during an experiment rather than in a separate calibration session. It is based on general assumptions about human eye and head movements in peripheral vision. Thus, since an infant's eyes and head are both initially fixed on a central point, both are in the straight-ahead position (0 degree). Next, when a target is shown approximately 30 degrees right or left in the field of vision, the eye is first directed to it. A head movement follows, as a result of which the eye is positioned over the target. To compensate for this overshoot, the eye moves backward in relation to the head. As a result, the eye eventually returns to its primary position with both eye and head together facing the target. Besides, in some trials, peripheral stimulus localization was accomplished almost exclusively with the eyes, while the head moved very little.

The calibration method of Koga et al. [15] makes the XY-tracker values – the second most frequent outputs – correspond to a visual angle of 30 degrees. Then, using these values, the so-called calibration scale factors, the XY-tracker outputs were linearly transformed into a visual angle. Here, it should be recalled that the result of the transformation is an absolute, not a relative, value.

Because in our research the infants can move their head freely, the eye position detected through the corneal reflection is an amalgam of the eye position in the orbit and the head position in space. To obtain the eye positions in the orbit or relative to the head, we subtract the head position outputs (which are a reflection of the chrome ball on the infant's forehead) from the corneal reflection outputs. The XY-tracker's outputs were linearly transformed into visual angles in terms of both the eye positions relative to the head and the head positions relative to space. Then, to compute the eye position relative to space (gaze) in a visual angle, the eye positions relative to the head and the head positions relative to space were added. As a result of these operations, we found the point in space where the eye gazed, and that position represents the amplitude of the gaze in a visual angle.

### Anti-saccade task

The procedure in the anti-saccade task (Figure 1a) followed that of Johnson [3]. The centering fixation stimuli were composed of brightly-colored moving abstract figures and subtended 5 degrees of the visual angle. The stimuli were accompanied by synchronized sounds. While the infant looked at the fixation, the experimenter pressed a key, which triggered presentation of the cue, a yellow triangle (3 degrees in width), on one of the two sides. The peripheral cue was presented for 100 ms together with the central fixation. Following the offset of both the central and cue stimuli, there was a 400-ms gap before presentation of the target on the side opposite that on which the cue had appeared. The target was composed of colored shapes moved in synchrony with simple sounds. The experiment consisted of a total of 32 trials, with 16 left and 16 right targets in pseudo-random order. The training phase consisted of the first five trials, after which the test phase of the experiment began.

### Inhibition-of-return task

The inhibition-of-return task procedure (Figure 1b) followed that of Butcher et al. [19]. Each trial began with the presentation of attractive centering fixation stimuli similar to those used in the anti-saccade task. As before, after the key was pressed, a peripheral cue appeared to the right or left of the fixation stimuli. The cue, a yellow triangle (3 degrees in width), was presented for 100 ms without the central fixation. As the cue disappeared, the central stimulus reappeared for 900 ms. Following offset of the central stimulus, the bilateral target was presented. The target was composed of moving colored abstract shapes associated with auditory signals, the two sides of which were always identical. The experiment consisted of a total of 32 trials, with 16 left and 16 right cues in pseudo-random order.

### Latencies

In the present study, only the horizontal component (x-axis) was analyzed from the two-dimensional coordination (horizontal and vertical outputs) produced by the X-Y tracker, allowing us to examine latencies, amplitudes and velocity.

We defined latency as the elapsed time between the sampling time at the cue presentation and the time at the maximum acceleration for each trial of each infant. We used latencies to investigate whether a head movement precedes an eye movement in younger infants.

The maximum acceleration is obtained through a series of calculations. First, to ascertain the velocity (*v*_*i*_) of a horizontal eye movement at the *i*th sampling time, we should know the gradient of the horizontal outputs (*x*_*i*_) at the *i*th sampling time. This gradient was computed by the formula (*x*_*i*+1 _- *x*_*i*-1_)/(2/30). Here, 2/30 stands for the elapsed time from the (*i *- 1)th sampling time to the (*i *+ 1)th one. This operation is equivalent to the first differential of the horizontal eye movement with respect to time. Next, by differentiation of the derived velocity, i.e., through the equation (*v*_*i*+1 _- *v*_*i*-1_)/(2/30), we obtain the acceleration. That series of operations is equivalent to the second differential of the horizontal eye movement with respect to time. Finally, we determine the maximal acceleration for each trial of each infant, and subtract the sampling time at the target presentation from the one at the maximum acceleration. As the calibrated data are a linear transformation of the raw data, it does not matter whether the latency is based on the calibrated data or the raw data for comparison of an intra-infant. We opted to compute latency based on the raw data.

## Results

### Trials for analysis

We mainly analyzed those trials in which the infant's gaze moved directly from fixation on the target locations, i.e., the side opposite the cue or the cued locations. Trials in which the gaze deviated from the direct line to those locations were not included in the analysis. Only responses occurring after the cue onset in the anti-saccade task and after the target onset in the inhibition-of-return task were included. Based on the videotape in which the stimuli were superimposed on video images of the eye movement (Figure 2), two observers not directly involved in the experiment judged whether or not the trial was adequate for our analysis. The coefficient of agreement between the two coders was .88. In some trials which were judged to be adequate, we failed to digitize the eye or head movements by the X-Y tracker and had to exclude them.

For the infant anti-saccade paradigm, to separate visually triggered eye movements to the target from ones that are prepared based on the cue, we tentatively established one criterion, i.e., responses were excluded when both eye and head latency were longer than 1100 ms. Namely, it is up to 600 ms post-target onset, which was defined based on the average of each infant's mean latency of responses toward the cued side (755.3 ms ± 146.6). Here the latency of each trial was defined as either a faster latency of the eye or head. That is, 600 ms is about the average latency minus 1 SD. Then, when either the eye or head latency was less than 1100 ms, we considered it not to be visually triggered.

After taking the steps mentioned above, the following numbers of responses were analyzed for each of the age groups. The average numbers of responses, which were directed to the side opposite the cue in the anti-saccade test phase, were 7.8 (SD = 2.4) for the younger-age group, 6.6 (SD = 4.1) for the middle-age group, and 10.5 (SD = 6.3) for the older-age group. The average numbers of responses, which were directed to the cued location in the anti-saccade training and test phases, were 9.1 (SD = 5.4) for the younger-age group, 10.6 (SD = 6.1) for the middle-age group, and 7.8 (SD = 4.4) for the older-age group. In the task for inhibition of return, average numbers of responses directed to the side opposite the cue were 13.6 (SD = 6.7) for the younger-age group, 8.0 (SD = 1.4) for the middle-age group, and 8.4 (SD = 3.6) for the older-age group. The average numbers of responses directed to the cued location were 8.8 (SD = 3.3) for the younger-age group, 9.8 (SD = 5.5) for the middle-age group, and 7.0 (SD = 4.6) for the older-age group. In any event, for each trial, it was difficult to obtain completely digitized data whose sampling time was 33.3 ms all in one trial round.

### Looking toward cued side vs. non-cued side in anti-saccade paradigm

The latencies of each condition are presented in Table 1 during the anti-saccade task. Average reaction times for each experimental condition were subjected to a four-way repeated measure analysis of variance (ANOVA) with the following factors: side (non-cued, cued), organ (eye, head), direction (leftward, rightward), and age (younger, middle, older). Only the main effect of the side was marginally significant (*F*_(1,11) _= *3.72*, p = .08). Hence, the average latency to the target side (846.0 ms, SD = 228.8) was longer than the average latency to the cued side (741.5 ms, SD = 121.8). The interaction between side and direction was significant (*F*_(1,11) _= *8.77*, p < .05). Responses which were directed to the side opposite the cue occurred earlier toward the right (788.5 ms) than toward the left (934.3 ms), while the latencies of the responses directed to the cued location were 703.1 ms to the left and 780.6 ms to the right.

**Table 1:** Mean latencies (SD in parentheses) of the saccades during task for anti-saccade (ms)

Side	Organ	Direction	Age (months)					
			
			3~5		6~8		9~11	
Non-Cued	Eye	Left	1004.8	(231.2)	965.2	(176.0)	813.2	(238.4)
		Right	821.5	(214.3)	939.7	(179.0)	766.3	(242.6)
	Head	Left	939.2	(137.4)	1066.3	(147.5)	817.5	(233.4)
		Right	834.1	(218.4)	753.2	(298.0)	616.2	(217.6)
Cued	Eye	Left	600.4	(140.5)	703.7	(142.7)	749.7	(215.2)
		Right	760.2	(213.5)	805.7	(61.4)	773.7	(42.6)
	Head	Left	623.5	(166.5)	738.4	(140.4)	803.2	(198.8)
		Right	794.4	(221.7)	781.8	(101.8)	767.7	(47.7)

**Figure 3:**
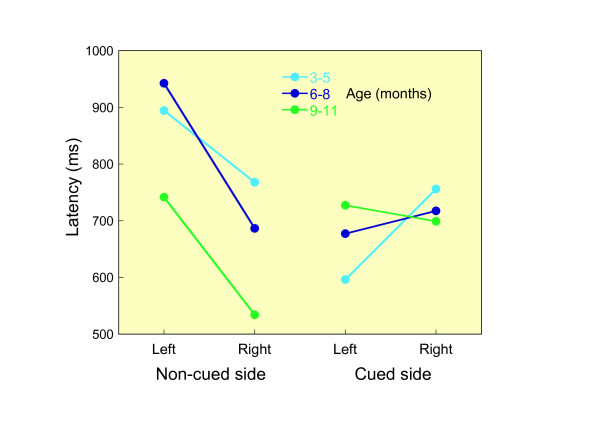
Mean latencies of each trial, which were defined as either a faster eye or head latency, to the right or left side, for non-cued and cued conditions in three age groups during anti-saccade task.

As a result of the ANOVA mentioned above, the main effect of the organ was not significant (*F*_(1,11) _= *.349*). Consequently, we defined the latency of each trial as either a faster latency of the eye or head and conducted a three-way repeated measure analysis of variance (ANOVA) with the following factors: side (non-cued, cued), direction (leftward, rightward), and age (younger, middle, older). These data were plotted in Figure 3. The main effect of the side was not significant (*F*_(1,11) _= *1.32*). However, the interaction between side and direction was significant (*F*_(1,11) _= *10.95*, p < .01). This interaction means that infants made faster saccade toward the side opposite the cue to the right than to the left (*F*_(1,21) _= *12.32*, p < .01), while there were no significant differences between the saccade to the cue to the left and to the right (*F*_(1,21) _= *0.636*). A similar asymmetry was reported with regard to the anticipatory responses of infants [20].

To ascertain the tendency of early age to initiate head movement fast, we categorized the responses of three age groups both to the non-cued and cued sides into three kinds according to the latencies of eye and head of each response: eye-first, head-first and same time. We applied a log-linear model to the age (3) × side (2) × organ (3) contingency table. As a result, the model including both an age × organ interaction and an age × side one best fits the table (χL2
 MathType@MTEF@5@5@+=feaafiart1ev1aaatCvAUfKttLearuWrP9MDH5MBPbIqV92AaeXatLxBI9gBaebbnrfifHhDYfgasaacH8akY=wiFfYdH8Gipec8Eeeu0xXdbba9frFj0=OqFfea0dXdd9vqai=hGuQ8kuc9pgc9s8qqaq=dirpe0xb9q8qiLsFr0=vr0=vr0dc8meaabaqaciaacaGaaeqabaqabeGadaaakeaaiiGacqWFhpWydaqhaaWcbaGaemitaWeabaGaeGOmaidaaaaa@30AA@ = 14.15, *df *= 6, *p *= .0279; *AIC *= 2188.78). The age × side interaction indicates that frequency of movements to the non-cued side is different from that to the cued one according to the age group. The age × organ interaction shows that the frequency of eye-first responses among the younger-age group is less than that in other age groups.

### Anti-saccade paradigm vs. inhibition-of-return paradigm: trajectories of eye and head movements

With regard to the reaction time data of the inhibition-of-return task (Table 2), ANOVA was applied with the following factors: side (non-cued, cued), organ (eye, head), direction (leftward, rightward), and age (younger, middle, older). The effect of side approached significance (*F*_(1,12) _= *4.09*, p = .066). Thus, the average latency to the non-cued side (586.2 ms, SD = 81.2) was faster than the average latency to the cued side (660.1 ms, SD = 120.8). Based on these data, we considered ourselves able to observe inhibition of return in our subjects. The interaction between organ and direction was significant (*F*_(1,12) _= *5.14*, p < .05). Toward the left, eye movements occurred earlier (605.8 ms) than head movements (651.0 ms), while the latencies of the movements directed to the right were 618.2 ms for the eye and 610.5 ms for the head. The faster leftward eye movements than head movements was specially observed in the response to the non-cued side, as the side × organ × direction interaction was marginally significant (*F*_(1,12) _= *3.75*, p = .077). That is, regarding the response side opposite the cue, eye movements occurred earlier (580.5 ms) than head movements (652.8 ms) toward the left, while the latencies of the movements directed to the right were 560.7 ms for the eye and 534.0 ms for the head. As for the responses to the cued side, the latencies of movements directed to the left were 631.1 ms for the eye and 649.3 ms for the head, while these toward the right were 675.7 ms for the eye and 686.9 ms for the head. The age × organ × direction interaction was also significant (*F*_(2,12) _= *5.01*, p < .05).

**Table 2:** Mean latencies (SD in parentheses) of the saccades during task for inhibition of return (ms)

Side	Organ	Direction	Age (months)					
			
			3~5		6~8		9~11	
Non-Cued	Eye	Left	565.1	(172.0)	635.8	(202.1)	540.7	(67.9)
		Right	543.1	(123.5)	600.4	(173.2)	538.8	(19.5)
	Head	Left	552.4	(164.7)	655.1	(198.9)	750.8	(246.6)
		Right	611.8	(109.0)	492.5	(155.5)	497.7	(170.1)
Cued	Eye	Left	707.3	(256.8)	563.8	(158.8)	622.2	(117.0)
		Right	639.7	(86.4)	683.7	(252.4)	703.6	(77.8)
	Head	Left	720.3	(262.7)	607.4	(189.9)	620.3	(130.3)
		Right	661.0	(74.4)	691.5	(255.3)	708.3	(38.1)

As for amplitude, the issue is where the eye and/or the head are inclined in space. Thus, to obtain the appropriate amplitude of the eye relative to space (gaze) and the head relative to space in a visual angle, calibration factors for each infant must be found through the calibration procedure [15]. However, for some infants, we could not obtain the calibration factors, because the second most frequent landing position was ambiguous. Consequently, one 6-month-old, one 7-month-old, one 10-month-old and one 11-month-old were excluded from analysis of amplitude after the calibration.

**Figure 4:**
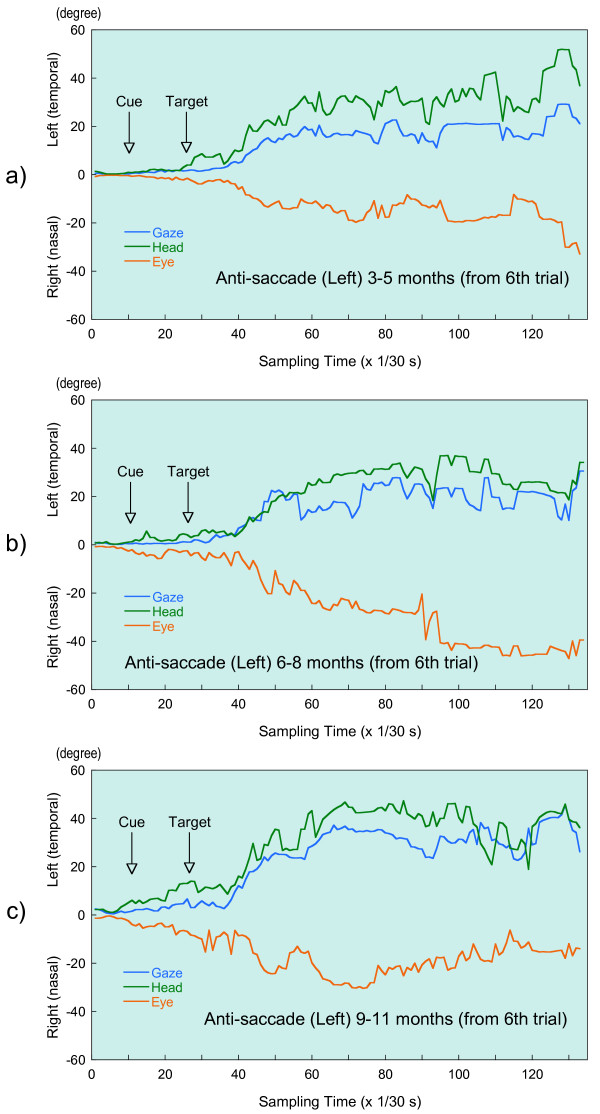
Illustration of eye-head gaze saccades during anti-saccade task in three age groups: a) 3–5 months, b) 6–8 months, C) 9–11 months. Traces of Gaze, Head and Eye are eye position in space, head position in space and eye position in head, respectively.

**Figure 5:**
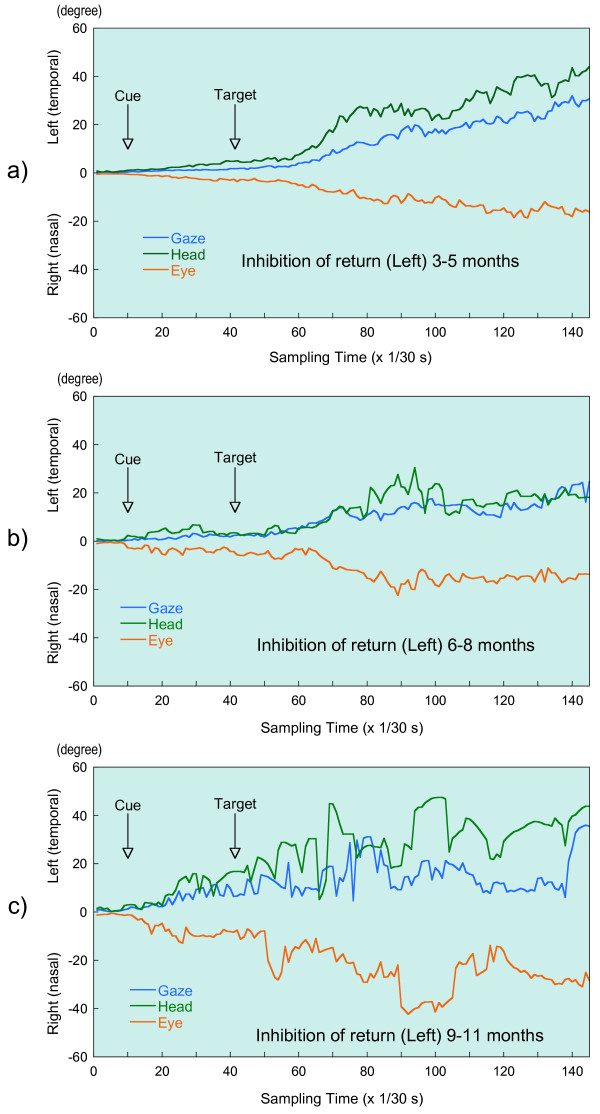
Illustration of eye-head gaze saccades during inhibition-of-return task in three age groups: a) 3–5 months, b) 6–8 months, C) 9–11 months. Traces of Gaze, Head and Eye are eye position in space, head position in space and eye position in head, respectively.

At each sampling time, for the eye relative to the head (eye), the head relative to the space (head) and the eye relative to the space (gaze), we calculated the harmonic mean of all available data of the infants whose calibration factors were determined. Figure 4 and 5 illustrate the harmonic mean of the eye, the head, and the gaze movements during task for anti-saccade and inhibition of return as a function of sampling times by age group, respectively. That is, y-axis in a visual angle stands for the amplitude. These saccades of both tasks are those which occurred in the leftward direction, opposite the cue, in the available trials.

In Figure 4 and 5 these movements begin around the sampling time for the target presentation, i.e., the 27^th ^sampling time for anti-saccade and the 43^rd ^for inhibition of return. From these target presentation times, the amplitude of gaze, head and eye continue to increase. Then these amplitudes appear to stabilize at around the 61^st ^sampling time for the anti-saccade and at about the 81^st ^sampling time for the inhibition of return. On the basis of Figure 4 and 5, we could assume steeper head movement during the anti-saccade task compared with those evoked during the task for inhibition of return in the youngest two age groups.

In Table 3 and 4, the maximal amplitudes of gaze, head and eye are presented by age group during two tasks, respectively. These amplitudes correspond to each fixed amplitude after the target presentation in Figure 4 and 5, although the data illustrated in Figure 4 and 5 are only for saccades occurring in the leftward direction. Hence, in the anti-saccade task, the mean amplitudes between the 61^st ^and the 90^th ^sampling time were calculated, while the mean value between the 81^st ^and 110^th ^sampling time were calculated for inhibition of return. Table 3 and 4 indicate that the youngest two subjects had smaller head movements in the inhibition-of-return task compared with those evoked in the anti-saccade task, while 9–11 month infants showed almost equal movements during the two tasks.

**Table 3:** Mean maximal amplitudes (SD in parentheses) of gaze, head and eye during task for anti-saccade of sampling time from 61 to 90 (deg)

		Age (months)					
		
		3~5		6~8		9~11	
Gaze	Left	17.2	(2.4)	20.6	(5.1)	33.2	(2.7)
	Right	-6.4	(1.1)	-5.9	(1.1)	-19.1	(1.6)
Head	Left	30.3	(3.1)	29.8	(2.3)	42.6	(3.0)
	Right	-29.5	(2.2)	-21.3	(5.7)	-31.1	(1.8)
Eye	Left	-14.2	(3.1)	-26.1	(2.1)	-25.5	(3.5)
	Right	36.6	(4.4)	16.2	(1.9)	26.5	(1.1)

Positive (negative) numbers mean left (right) direction.

**Table 4:** Mean maximal amplitudes (SD in parentheses) of gaze, head and eye during task for inhibition of return of sampling time from 81 to 110 (deg)

		Age (months)					
		
		3~5		6~8		9~11	
Gaze	Left	16.8	(2.4)	14.0	(1.9)	28.3	(6.1)
	Right	-6.3	(0.8)	-7.5	(1.1)	-27.9	(4.3)
Head	Left	25.6	(2.2)	19.6	(5.0)	47.4	(6.1)
	Right	-19.5	(2.4)	-19.8	(2.7)	-24.3	(2.6)
Eye	Left	-11.5	(1.4)	-16.1	(2.5)	-20.9	(3.0)
	Right	21.3	(2.1)	12.5	(3.2)	25.3	(1.4)

Positive (negative) numbers mean left (right) direction.

To explore the mean speed of gaze, head and eye from the sampling time at the target presentation to the one at the maximal amplitudes, we calculated a linear regression equation between amplitude and the sampling time and found a regression weight (or slope), which we take to represent the mean speed or velocity. In Table 5 and 6, the velocity of gaze, head and eye is calculated by age group during two tasks. In the task for anti-saccade, velocities were based on data between 27 and 60 sampling times, while for inhibition of return they were based on data between 43 and 80 sampling times. Table 5 and 6 also indicate that in the inhibition-of-return task the youngest two subjects showed smaller velocity of head movement compared to those in the anti-saccade task, although the head velocities of 9–11 month infants did not differ between the two tasks.

**Table 5:** Average velocity (deg/s) of gaze, head and eye during task for anti-saccade (sampling time: 27 to 60)

		Age (months)		
		
		3~5	6~8	9~11
Gaze	Left	18.9	18.9	26.6
	Right	-3.0	-0.09	-12.9
Head	Left	25.6	21.6	28.2
	Right	-24.6	-9.3	-25.3
Eye	Left	-12.2	-18.4	-11.0
	Right	36.5	6.7	21.9

Positive (negative) numbers mean left (right) direction

**Table 6:** Average velocity (deg/s) of gaze, head and eye during task for inhibition of return (sampling time: 43 to 80)

		Age (months)		
		
		3~5	6~8	9~11
Gaze	Left	9.5	9.1	22.3
	Right	-3.8	-3.2	-13.7
Head	Left	20.6	9.9	39.4
	Right	-17.6	-12.5	-18.7
Eye	Left	-6.3	-6.9	-7.8
	Right	18.0	10.5	19.6

Positive (negative) numbers mean left (right) direction

The relationship between eye and head movements during these tasks was also discussed in light of the categorical data mentioned above. We categorized the responses of the three age groups to the non-cued sides during the two tasks into three kinds of responses: eye-first, head-first and same time. Then we applied a log-linear model to the age (3) × task (2: Anti-saccade vs. Inhibition of return) × organ (3) contingency table. As a result, the model including both an age × task interaction and an age × organ interaction is deemed to best fit the table (χL2
 MathType@MTEF@5@5@+=feaafiart1ev1aaatCvAUfKttLearuWrP9MDH5MBPbIqV92AaeXatLxBI9gBaebbnrfifHhDYfgasaacH8akY=wiFfYdH8Gipec8Eeeu0xXdbba9frFj0=OqFfea0dXdd9vqai=hGuQ8kuc9pgc9s8qqaq=dirpe0xb9q8qiLsFr0=vr0=vr0dc8meaabaqaciaacaGaaeqabaqabeGadaaakeaaiiGacqWFhpWydaqhaaWcbaGaemitaWeabaGaeGOmaidaaaaa@30AA@ = 2.46, *df *= 6, *p *= .8727; *AIC *= 2184.51). The age × task interaction is beside the point, because it shows that each age group has a different sample size. The age × organ interaction indicates that the frequency of eye-first responses among the younger-age group is less than that among other groups, and that the frequency of head-first response among the low- and middle-age groups is higher than that of the older-age group.

## Discussion

The present study sought to investigate the characteristics of infant eye and head movements during an anti-saccade task. For this purpose, first, we compared the responses toward the side opposite the cue with the responses to the cued location in the anti-saccade task. Second, we attempted to a quantitatively analyze infant eye and head movements toward the side opposite the cue during tasks for anti-saccades and inhibition of return. The comparison is made to determine whether the inhibition of return shows signs of maturity well before the anti-saccade task, since the superior colliculus involved in the inhibition of return is thought to develop much earlier in life than the frontal lobes.

### Latencies of eye and head movements in infants

In general, the present eccentricity (30 deg) was accomplished with eye and head movements. With regard to the latencies of eye and head, younger infants less often initiated eye movement. These findings are consistent with previous infant data [6,14,21]. On the other hand, human adult subjects sometimes reportedly move their head in the direction of the target before the gaze shift begins. This might be a distinguishing feature of the "predictive" mode of gaze shifts [22], whereas generally head movements should follow saccade onset [23]. However, the present infant results are convincing from a phylogenetical point of view in that a combined eye-head movement may represent an older system, which is designed to change the direction of gaze, but not to foveate any particular targets, while the eye saccade could be a more recent system designed to bring a visual target onto the fovea [12]. Ontogenically, eye-head gaze emerges in an early stage. Animals, like the rabbit, who have no fovea to align with the visual target, rarely make saccades without making head movements [24]. Also in the cat, eye and head movements are closely related, as gaze control in the cat is finer than in the rabbit [25].

### Infant responses during anti-saccade task

In the present study of the anti-saccade task, to exclude the measured movements to the non-cued side of visual guidance, we employed our own criterion without using the conventional adult criterion (i.e., for a response longer than 100 ms after target presentation). Thus, we regard responses longer than 600 ms post-target onset as visually triggered responses. There are two reasons for this criterion: 1) The method used to measure the response time and perform calculations in this study differed from the conventional research approach with adult subjects, in which the head of the participant is stabilized or electrooculography is applied. In head-free condition, variations in latency between eye and head movements, which is dependent on target eccentricity, predictability etc., have been shown in human [26]. How gaze control is allocated into separate commands for eye and head movement is still unclear [27]; and 2) Describing the response dynamics of infants was the key objective of the present study. We thus sought to analyze reactions other than those that were clearly visually triggered. As a result, the latencies of responses toward the side opposite the cue were marginally longer than those of responses toward the cued side, as found in previous infant [4] and adult studies [1,5,10,11]. This difference in latency is possibly attributable to the responses toward the cued side, being an exogenously driven response, whereas the anti-saccades are endogenously controlled and require more computational resources [3]. Hence, an infant might compute the goal of his/her movement not from the visual trigger but from an internal source. This is consistent with the previous study that 4-month-old infants readily make anticipatory saccades [20,28]. As for the asymmetry of the anticipatory saccades of infants, our findings corresponded to those of Csibra et al. [20], i.e., the response was fast toward the right. Fischer et al. [9] and Munoz et al. [10], investigating anti-saccade task performances between ages 8–70 years and 5–79 years respectively, also noted the same trend in asymmetry.

To confirm that these are really anti-saccades and not saccades toward the target, we should include the latencies in a comparable task with no cue. And the experiment using the same condition should be applied to the same infants except that the cue-target time interval is increased, following Guitton et al. [5]. Scerif et al. [4] chose 700 ms for the cue-target time interval with reference to Guitton et al. [5], and on the basis of 800 ms (to be specific, up to 100 ms post-target onset) distinguished between anti-saccades and reactive saccades, which are stimulus driven, rather than anticipatory. Although almost half of target looks were classified as reactive saccades in Scerif et al. [4], these may well include infant anticipatory responses.

### Performance during anti-saccade paradigm vs. inhibition-of-return paradigm

When we compare the responses toward the side opposite the cue during the inhibition-of-return task with anti-saccades, one of the findings is that infants tend to move eyes before their head towards the left in the inhibition-of-return task. No such difference was observed between eye and head movements in the anti-saccade task. Since we found that younger infants tend to move their head before their eyes, the difference between the two tasks mentioned above could suggest the faster development of inhibition-of-return performances, and this would confirm the idea of inhibition of return being a phylogenetically ancient midbrain mechanism [17,29]. Previous study showed that inhibition of return reaches near-adult levels by 6 months [30], while the number of anti-saccades increased greatly during the toddler years [4].

Based on Figure 4 and 5, infants in all three age groups made steeper head movements in the anti-saccade task, while in the inhibition-of-return task we observed smooth head movements especially in the younger two ages. In other words, infants with the younger two ages made gentle head movement in the inhibition-of-return task, although they could shift their head sharply with large amplitude. First, we thought that it could be related to the differences in the target condition. That is, in the inhibition-of-return task, subjects were given targets in both the right and left visual fields, while in the anti-saccade task, a target was presented only on the side opposite from that in which the cue had appeared. In this regard, in the study of the developmental course of inhibition of return in 3- and 6-month-old babies, Clohessy et al. [30] examined the effect of whether the display had only one unilateral or bilateral target. They noted that although there are some differences of reaction and movement times between the unilateral and bilateral trials, these differences are quite small. Movement time was defined as the time between the subject's initial response and the time at which he or she was fixated on the periphery. As this movement time could be related to the gradient of movements in Figure 5, investigators' results suggest a rather small effect of unilateral or bilateral target on the present difference observed in head movement.

Another difference of experimental conditions could be whether there is gap before the target presentation. Thus, in the anti-saccade task, the fixation and cue disappeared 400 ms before the appearance of the eccentric target. On the other hand, in the inhibition-of-return task, the central fixation was replaced by targets. In infants, gap conditions produced the fastest saccadic latencies compared to the no-overlap and overlap conditions [8,31]. Thus far, since no one has noted the gap effect on the head movement, it is not worth discussing observed differences along this line.

Next, we speculate on the difference of neural substrates between anti-saccade and inhibition-of-return tasks. Cowie & Robinson [32], using electrical stimulation of Macaques, demonstrate that both the superior colliculus and gigantocellular reticular nucleus mediate head movements during gaze shifts. The collicular head movements were predominantly associated with gaze shifts, while stimulation of the medullary reticular region produced ipsilateral head movements with no shift of gaze. It is supposed that head movement from the medullary reticular site plays a role in several forms of head movement, such as those which are concerned with postural reflexes, started volitionally, and/or oriented to external events. We speculate that present head movements in the inhibition of return might correspond to the collicular head movements with the gaze shifts, since inhibition of return could be strongly linked to the eye movement system of the superior colliculus [17,29]. On the other hand, in the anti-saccade task, the medullary reticular site contributes more to the head movements than in the inhibition-of-return task. Signals which emerge from the medullary reticular region lead to fast and reproducible movements of the head [32]. Hence, although the oculomotor system for anti-saccade is late-maturing, unlike in the inhibition-of-return task, from early infancy a direct command to the head is possible. Thus, it would seem surprising if an infant of a younger age were to show sharp head movements when shifting gazes. One view is that young children (< 8 years of age), who reportedly have difficulty in the anti-saccade task [18], might accomplish anti-saccades more easily in company with head movements.

### Left/right asymmetry

Lastly, we comment on the left/right asymmetry effects observed in turning behaviors. Anti-saccades occurred faster towards the right than the left, while in the inhibition-of-return task the infant made faster leftward eye movements than head movements in the same direction. The observed asymmetry during the anti-saccade task could be compatible with the physiological rightward bias in triggering eye movements [33-35]. For example, Sheliga et al. [33] found that the trajectory of the saccades deviated contralateral to the hemifield of stimulus presentation, and this deviation was larger to the right. On the other hand, the leftward bias of eye movements observed during the inhibition-of-return task could be related to the right lateralized ventral frontoparietal network, which might involve reorienting of attention to an unattended location or a shift of attention from a cued location to an uncued one [36]. Moreover, decreased performance was observed only when transcranial magnetic stimulation (TMS) was applied over the right frontal eye field and only when the cue was invalid, namely, when attention had to be disengaged and shifted to the opposite hemifield [37]. Here, it is speculated that TMS over this region might cause the disruption in shifting attention similar to the way it disrupts eye movement preparation. This speculation and the functional asymmetry observed for interference with shifts of attention might support the present leftward bias of eye movements in the inhibition-of-return task. The reason for these evident asymmetries remains unknown.

## Conclusion

We attempted to quantitatively examine the infant's eye and head movements in response toward the side opposite the cue during the anti-saccade and inhibition-of-return tasks in the head-free condition. In the anti-saccade paradigm, based on the response in accord with our criteria, the tendency was to a greater latency with a response to the non-cued side. Moreover, these responses were faster toward the right, which is a result consistent with previous studies in children and adults.

We confirmed that younger infants move their head and eyes together, often starting with the head in both tasks. However, regarding the leftward movements during inhibition-of-return task, the latency of eye was smaller than that of head. This kind of difference in latency was not observed between eye and head movements in the anti-saccade task. Besides, head movements of responses observed in the anti-saccade task looked steeper than those observed in the inhibition-of-return task. We consider that these differences between the two tasks were because inhibition of return is based on an old, earlier developing neural system. However, our discussion is quite speculative in the development phase, so further research is warranted to verify these preliminary findings.

## Competing interests

The author(s) declare that they have no competing interests.

## Authors' contributions

AN conceived the study. AN and MS conducted the experiments. MS performed analyses and AN prepared the initial draft of the manuscript. All authors approved the final manuscript.

## Supplementary Material

Additional file 1 Video record of 6-month infant during the anti-saccade task. Upper white dot is reflection of the small steel ball bearing representing head movements. Bottom two dots are corneal reflections; right one represents the left eye. These were digitized by an X-Y tracker off-line. Click here for file
